# Perceived knowledge, attitudes, and barriers toward telemedicine adoption Among adults in Mogadishu, Somalia: a cross-sectional study

**DOI:** 10.3389/fdgth.2026.1963595

**Published:** 2026-10-12

**Authors:** Mohamed Sharif Abdi, Sayidcali Warsame Alinuur, Hodan Abdullahi Hassan, Maslah Kamal Mohamed, Yusuf Hared Abdi, Naciimo Cabdishakuur Cisman, Zeinab Mohamed Ahmed, Yakub Burhan Abdullahi, Sharmake Gaiye Bashir, Naima Ibrahim Ahmed, Gallad D. Hassan

**Affiliations:** 1 Center for Health Research and Innovation, Somali National University, Mogadishu, Somalia; 2 Faculty of Health Science, Salaam University, Mogadishu, Somalia; 3 Faculty of Health Sciences and Tropical Medicine, Somali National University, Mogadishu, Somalia; 4 Faculty of Medicine, Somali National University, Mogadishu, Somalia; 5 Department of Public Health, Faculty of Graduate Studies and Research Somali National University, Mogadishu, Somalia

**Keywords:** attitudes, digital health, knowledge, Somalia, telehealth adoption, telemedicine

## Abstract

**Background:**

Telemedicine has emerged as an important strategy for improving access to healthcare, particularly in fragile health systems facing shortages of specialists and significant geographic barriers. Despite its potential, evidence on community readiness for telemedicine adoption in Somalia remains limited.

**Methods:**

A community-based cross-sectional study was conducted between February and April 2026 among 425 adults in Mogadishu. Participants were recruited using convenience and snowball sampling through social media platforms. Data were collected using a structured questionnaire assessing telemedicine knowledge, attitudes, and perceived barriers. Descriptive statistics summarized participant responses, while multivariable linear regression identified independent predictors of knowledge and attitude scores.

**Results:**

The mean knowledge score indicated limited-to-moderate knowledge across the five-item measure. Although 60.9% had previously heard of telemedicine, fewer than half had observed (44.0%) or used (44.7%) telemedicine services. Attitudes toward telemedicine were generally favorable, with mean item scores ranging from 3.21 to 3.42 on a five-point scale. The most frequently perceived barriers were preference for face-to-face consultations, technological limitations, and patient resistance. Higher knowledge scores were independently associated with age, income, chronic illness, transportation-cost barriers, and social media use, whereas positive attitudes were independently associated with marital status, educational attainment, and smartphone ownership.

**Conclusions:**

Adults in Mogadishu demonstrate moderate knowledge and generally positive attitudes toward telemedicine despite persistent technological and behavioral barriers. Expanding digital literacy, strengthening digital infrastructure, and establishing supportive regulatory and implementation frameworks will be essential to facilitate equitable and sustainable telemedicine integration into Somalia's health system.

## 1 Introduction

Telemedicine has evolved from a niche clinical tool into a central pillar of modern health systems, driven by advances in mobile connectivity, broadband penetration, and policy commitment from global health bodies (1). The World Health Organization formalized this shift through its Global Strategy on Digital Health 2020–2025, which frames digital health technologies as instruments for strengthening health systems and advancing universal health coverage (2). This strategic vision has since been extended through 2027, reflecting sustained international consensus on digital health as a long-term structural priority rather than a transient pandemic-era innovation (3).

The COVID-19 pandemic acted as a catalytic force for telemedicine, compelling health systems worldwide to rapidly substitute in-person consultations with remote care modalities such as video conferencing, phone triage, and asynchronous messaging (4). A systematic review of telemedicine facilitators and barriers during the first year of the pandemic found that the majority of studies reported high patient satisfaction and comparable or improved clinical outcomes relative to in-person care, reinforcing telemedicine's clinical legitimacy beyond emergency substitution (5). Reported benefits span reduced patient travel burden, decreased exposure to infectious pathogens, improved continuity of chronic disease management, and more efficient use of scarce specialist time (4). These accumulated efficiencies have sustained global adoption momentum well past the acute pandemic phase, with health systems increasingly institutionalizing telemedicine within routine care pathways rather than treating it as a temporary contingency (1).

In low- and middle-income countries, telemedicine addresses a fundamentally different set of constraints than in high-income settings, chiefly severe health workforce shortages and vast geographic distances separating patients from specialist care (6). A focused-on South Africa, Kenya, and Nigeria found that these three countries lead Sub-Saharan Africa in telemedicine adoption, with applications ranging from specialist teleconsultation and chronic disease management to mobile-health-supported maternal and HIV care (6). Despite this progress, the same review identified persistent regulatory gaps, limited digital literacy, and inadequate broadband infrastructure as recurring implementation barriers across the region, underscoring that technological availability alone does not guarantee adoption (6). Complementary evidence from LMIC-focused reviews of telemedicine during COVID-19 similarly emphasizes that sustainability depends on multisectoral partnerships, health informatics investment, and community-level readiness assessments rather than technology deployment in isolation (7).

Armed conflict and humanitarian crises routinely disrupt formal healthcare infrastructure, displacing health workers, damaging facilities, and severing patient access to specialist services precisely when population health needs intensify (8). Telemedicine interventions evaluated across six conflict-affected countries in the WHO Eastern Mediterranean Region demonstrated feasibility using low-cost, accessible technologies, suggesting that even minimally resourced settings can sustain remote care delivery when appropriately designed (8). A narrative review of eHealth applications in contemporary conflict settings similarly concluded that digital tools help bridge geographic, epidemiologic, and clinical service gaps created by insecurity and population displacement (9). The wartime Sudan reinforces this potential, documenting meaningful teleconsultation utilization among internally displaced persons as a mechanism for maintaining care continuity amid healthcare system collapse (10). Nonetheless, the same body of evidence highlights persistent concerns around data privacy, cultural and linguistic barriers, and limited health literacy that must be addressed for digital health interventions to be genuinely accessible in fragile contexts (10).

Somalia's health system remains characterized by chronic underfunding, fragmented governance following decades of conflict, and a workforce far below international benchmarks, with nurse, midwife, and doctor densities under four per 10,000 population against a WHO-recommended threshold of 23 (11). This shortage of specialists is compounded by a pronounced urban-rural disparity, with the majority of the country's 112 identified health professions schools and associated skilled personnel concentrated in urban centers such as Benadir, leaving rural and nomadic populations with severely constrained access to qualified care (12). These structural deficits create substantial access barriers, particularly for populations requiring specialist consultation unavailable outside major cities (1). Against this backdrop, Somalia has experienced notable growth in mobile phone and internet connectivity, although the country's Digital Inclusion Policy acknowledges that nearly 85 percent of the population still lacks access to modern digital services, reflecting a persistent digital divide despite expanding mobile network coverage (1). National authorities have begun incorporating digital innovations, including telemedicine-oriented initiatives, into broader health system strengthening efforts aimed at improving access and quality of care (1).

Existing Somali research on digital health has concentrated predominantly on eHealth system architecture, health information systems, and mobile health applications for disease surveillance and service delivery (13). Comparable population-level assessments from other settings, such as a national survey evaluating knowledge, attitudes, and barriers toward telemedicine among the general Egyptian population, illustrate the type of evidence that remains largely absent for Somalia (14). Consequently, little is currently known about public awareness, knowledge, attitudes, perceived barriers, and willingness to adopt telemedicine among the general adult population in Somalia, despite the country's documented specialist shortages and urban-rural access disparities that would plausibly benefit from expanded telemedicine uptake (15). This gap limits the ability of policymakers and implementers to design telemedicine programs that are responsive to actual community readiness and concerns rather than assumptions drawn from health-system-level data alone (15).

This study aims to assess perceived knowledge, attitudes, and barriers toward telemedicine adoption among adults in Mogadishu, Somalia. Specifically, the study seeks to assess community knowledge regarding telemedicine, examine attitudes toward its adoption, and identify the perceived barriers that may hinder uptake. It further aims to determine the sociodemographic factors associated with telemedicine knowledge, identify factors associated with positive attitudes toward telemedicine, and explore how healthcare access and digital access factors relate to telemedicine perceptions among the study population.

## 2 Methods

### 2.1 Study design and sampling

This study used a community-based, cross-sectional, survey-based research design conducted between February and April 2026 among adults residing in Mogadishu, Somalia. The study was conducted in a web-based, anonymous, and voluntary manner. Because the target population was considered large and the exact number of eligible adults residing in Mogadishu was unknown, the sample size was calculated using the standard single-population proportion formula for an unknown population. Based on a 95% confidence level, a 5% margin of error, and a response distribution of 50%, the minimum required sample size was 384 participants. To compensate for potential incomplete responses and improve the precision of the estimates, an additional 10% was added, resulting in a target sample size of 425 participants, all of whom completed the survey and were included in the final analysis. The target population included adults aged 18 years and older residing in Mogadishu without restrictions regarding sex, marital status, educational level, employment status, or income. Participants with incomplete questionnaires were excluded from the final analysis.

Two non-probability sampling techniques were employed. First, convenience sampling was used, whereby participants were recruited through a direct survey link shared via WhatsApp. Second, a simplified snowball sampling technique was applied by encouraging participants to forward the survey link to other eligible adults within their social networks. The survey link was distributed through several social media platforms, including WhatsApp, Facebook, Telegram, and other online community groups. Because the survey link was distributed through online networks and individual-level recruitment exposure was not tracked, the number of individuals who received, viewed, or declined participation was not available. Therefore, a conventional response or non-response rate could not be calculated. Participation was voluntary and anonymous. By agreeing to participate in the survey, respondents provided implied informed consent before completing the questionnaire. No incentives were provided for participation.

### 2.2 Measurement tool and data collection

The questionnaire was not a previously validated instrument. It was developed following an extensive review of the telemedicine literature, with several items adapted from a previously published telemedicine survey (16), and modified to reflect the Somali context. The English version was translated into Somali using forward and backward translation to improve semantic equivalence.

Before the main survey, the questionnaire was pilot-tested among 20 adults from the target population to assess clarity, comprehensibility, and appropriateness of wording. Minor modifications were made based on participants’ feedback. Internal consistency reliability was assessed using Cronbach's alpha coefficient. The reliability coefficients obtained for the knowledge, attitude, and barrier domains were 0.72, 0.84, and 0.79, respectively.

The questionnaire consisted of five sections: (1) sociodemographic characteristics (7 items); (2) health and digital access characteristics (7 items); (3) knowledge of telemedicine (5 items); (4) perceived attitudes toward telemedicine adoption (7 items); and (5) perceived barriers to telemedicine adoption (6 items). Knowledge items were measured using dichotomous (Yes/No) responses, whereas attitude and barrier items were measured using a five-point Likert scale, where 1 = strongly disagree, 2 = disagree, 3 = neutral, 4 = agree, and 5 = strongly agree.

The survey was administered electronically using KoBoToolbox, and the survey link was distributed through social media platforms, including WhatsApp, Facebook, and Telegram, as well as other online community groups. Participation was voluntary and anonymous, and informed consent was obtained before completion of the questionnaire. Data were exported to Microsoft Excel for data cleaning and coding before statistical analysis.

### 2.3 Independent variables

For sociodemographic variables, age was categorized into five groups: 18–29 years (reference category), 30–39 years, 40–49 years, 50–59 years, and ≥60 years. Sex was coded as 1 for male and 0 for female (reference category). Marital status was captured as a binary variable, with 1 representing married and 0 representing single (reference category). Educational level was categorized into four groups: no formal education (reference category), primary education, secondary education, and university education or above. Employment status was coded as 1 for employed and 0 for unemployed (reference category). Family size was categorized as ≤5 (reference category) and ≥6 members. Monthly income was categorized into low (reference category), middle, and high.

Health and digital-access variables included previous health facility visits, chronic illness requiring follow-up, distance as a barrier to healthcare access, transportation cost as a barrier to healthcare access, smartphone ownership, regular internet access, and social media account ownership. These variables were coded as binary variables (0 = No and 1 = Yes).

### 2.4 Dependent variables

For the knowledge questions (*n* = 5), scores of 0 and 1 were assigned to “No” and “Yes” responses, respectively, resulting in a total score ranging from 0 to 5. The perceived attitude (*n* = 7) and perceived barrier (*n* = 6) items were measured using a five-point Likert scale, yielding total scores ranging from 7 to 35 and 6–30, respectively. Responses of strongly disagree, disagree, neutral, agree, and strongly agree were assigned numerical values of 1, 2, 3, 4, and 5, respectively, during statistical analysis. Higher scores indicated better knowledge of telemedicine, more positive attitudes toward telemedicine adoption, and stronger perceived barriers.

### 2.5 Analysis methods

Data were analyzed using Stata version 17 Categorical variables were summarized using frequencies and percentages, whereas continuous variables were presented as means and standard deviations (SDs). For each knowledge item, endorsement by more than 50% of respondents was used only to describe the item-level response pattern and was not used to define an overall poor, moderate, or good knowledge category. Because no validated overall knowledge cut-off was established for the questionnaire, the total knowledge score was analyzed as a continuous variable ranging from 0 to 5.

Knowledge, attitude, and barrier scores were summarized using descriptive statistics. Independent-samples Student's t-tests were used to compare mean scores between variables with two categories, whereas one-way analysis of variance (ANOVA) was used for variables with three or more categories. Multivariable linear regression analysis was performed to identify factors independently associated with knowledge and attitude scores. Ordinary least squares regression was used after creating dummy variables for categorical predictors. The assumptions of linear regression were assessed, including linearity, homoscedasticity, approximate normality of residuals, independence of observations, and the presence of influential observations. Residual-versus-fitted plots and normal probability plots were used to assess model assumptions, and influential observations were evaluated using standard regression diagnostics.

Multicollinearity among independent variables was assessed using variance inflation factors (VIFs). A VIF value below 5 was considered to indicate no substantial multicollinearity. Model feasibility was considered in relation to the sample size and number of estimated parameters. Each multivariable model included 425 observations and 17 estimated slope parameters, providing approximately 25 observations per estimated parameter.

Regression coefficients (*β*), 95% confidence intervals (CIs), and two-sided *P* values were reported for all variables, with prespecified reference categories for categorical predictors. Statistical significance was determined using a two-sided *P*-value <0.05. Overall model fit was evaluated using the coefficient of determination (R^2^), adjusted R^2^, the root mean squared error, and the overall model *F* test with its corresponding *P* value.

## 3 Result

Table 1 presents the sociodemographic profile of the 425 respondents, revealing a predominantly young, educated, and digitally connected population. The majority of participants were aged 18–29 years (54.82%), single (76.47%), and had attained university-level education (62.35%), while the sample was nearly evenly split by sex (51.06% female, 48.94% male). Over half of respondents were unemployed (58.35%) and reported middle-level income (54.12%), with most living in households of five or fewer members (70.12%). Notably, a substantial proportion had prior contact with the health system, having visited a health facility before (78.82%), although a smaller subset reported chronic illness requiring follow-up (33.65%); meanwhile, distance (62.35%) and transport cost (63.53%) were commonly cited as barriers to healthcare access, underscoring persistent structural constraints to service utilization. Among respondents, smartphone ownership was 87.29%, regular internet access was 79.53%, and social media account ownership was 84.00%. These proportions describe the characteristics of the surveyed sample and should not be interpreted as population-representative estimates for adults in Mogadishu because recruitment relied on online convenience and snowball sampling.

**Table 1:** Sociodemographic characteristics of respondents (*N* = 425).

Variable	Category	Frequency (*n*)	Percentage (%)
Age Group	18–29 years	233	54.82
	30–39 years	103	24.24
	40–49 years	30	7.06
	50–59 years	42	9.88
	≥60 years	17	4.00
Sex	Female	217	51.06
	Male	208	48.94
Marital Status	Single	325	76.47
	Married	100	23.53
Educational Level	No formal education	52	12.24
	Primary education	33	7.76
	Secondary education	75	17.65
	University and above	265	62.35
Employment Status	Employed	177	41.65
	Unemployed	248	58.35
Number of Family Members	≤5	298	70.12
	≥6	127	29.88
Average Monthly Income	Low	145	34.12
	Middle	230	54.12
	High	50	11.76
Visited a Health Facility Before	Yes	335	78.82
	No	90	21.18
Chronic Illness Requiring Follow-up	Yes	143	33.65
	No	282	66.35
Distance a Barrier to Healthcare Access	Yes	265	62.35
	No	160	37.65
Transport Cost a Barrier to Healthcare Access	Yes	270	63.53
	No	155	36.47
Ownership of a Smartphone	Yes	371	87.29
	No	54	12.71
Regular Internet Access	Yes	338	79.53
	No	87	20.47
Social Media Account	Yes	357	84.00
	No	68	16.00

Table 2 summarizes mean knowledge and attitude scores across participant characteristics. Mean knowledge scores were higher among respondents aged 30–39 years and ≥60 years than among those aged 18–29 years, and were higher among married, employed, higher-income, previously healthcare-exposed, chronically ill, and digitally connected respondents. Knowledge scores were also higher among respondents reporting distance or transportation-cost barriers. Attitude scores were higher among married respondents and those with secondary education, and were also higher among respondents with larger households and access to smartphones, regular internet, and social media. Differences by sex were small for both outcomes.

**Table 2:** Comparison of the mean scores of knowledges and attitudes according to the sociodemographic characteristics (*N* = 425).

Variable	Participant, *n* (%)	Knowledge score		Attitude score	
		Value, mean (SD)	*P* value	Value, mean (SD)	*P* value
Age group			<0.001		0.004
18–29 years	233 (54.8)	2.53 (1.65)		22.61 (5.84)	
30–39 years	103 (24.2)	3.41 (1.49)		25.04 (7.04)	
40–49 years	30 (7.1)	3.37 (1.38)		24.40 (7.39)	
50–59 years	42 (9.9)	3.02 (1.68)		23.29 (7.59)	
≥ 60 years	17 (4.0)	4.06 (0.75)		26.76 (2.71)	
Sex			0.652		0.594
Female	217 (51.1)	2.88 (1.63)		23.40 (6.41)	
Male	208 (48.9)	2.95 (1.63)		23.73 (6.52)	
Marital status			0.002		<0.001
Single	325 (76.5)	2.77 (1.67)		22.90 (6.24)	
Married	100 (23.5)	3.36 (1.43)		25.72 (6.71)	
Educational level			0.185		0.038
No formal education	52 (12.2)	3.33 (1.76)		22.44 (8.47)	
Primary education	33 (7.8)	3.06 (1.69)		21.85 (7.25)	
Secondary education	75 (17.6)	2.93 (1.55)		25.15 (6.39)	
University and above	265 (62.4)	2.80 (1.61)		23.54 (5.82)	
Employment status			<0.001		0.362
Unemployed	248 (58.4)	2.60 (1.57)		23.32 (5.72)	
Employed	177 (41.6)	3.35 (1.61)		23.90 (7.37)	
Number of family members			0.828		0.042
≤ 5	298 (70.1)	2.90 (1.62)		23.14 (6.69)	
≥ 6	127 (29.9)	2.94 (1.66)		24.54 (5.77)	
Average monthly income			<0.001		0.553
Low	145 (34.1)	2.31 (1.57)		23.38 (6.51)	
Middle	230 (54.1)	3.22 (1.59)		23.83 (6.03)	
High	50 (11.8)	3.22 (1.50)		22.82 (8.06)	
Visited a health facility before			<0.001		<0.001
No	90 (21.2)	1.81 (1.56)		21.36 (5.85)	
Yes	335 (78.8)	3.21 (1.52)		24.15 (6.49)	
Chronic illness			<0.001		0.692
No	282 (66.4)	2.61 (1.64)		23.47 (6.06)	
Yes	143 (33.6)	3.51 (1.44)		23.73 (7.20)	
Distance a barrier			<0.001		<0.001
No	160 (37.6)	1.99 (1.54)		21.32 (5.83)	
Yes	265 (62.4)	3.47 (1.42)		24.91 (6.45)	
Transport cost a barrier			<0.001		<0.001
No	155 (36.5)	2.05 (1.51)		21.98 (5.37)	
Yes	270 (63.5)	3.41 (1.48)		24.47 (6.85)	
Availability of smartphone			<0.001		<0.001
No	54 (12.7)	1.93 (1.67)		19.17 (6.46)	
Yes	371 (87.3)	3.05 (1.58)		24.20 (6.21)	
Regular internet access			<0.001		0.002
No	87 (20.5)	2.17 (1.61)		21.62 (6.48)	
Yes	338 (79.5)	3.10 (1.58)		24.06 (6.36)	
Availability of social media account			<0.001		0.003
No	68 (16.0)	2.03 (1.73)		21.47 (6.63)	
Yes	357 (84.0)	3.08 (1.56)		23.96 (6.35)	

Table 3 shows variable levels of telemedicine knowledge and prior exposure among respondents. Most had heard of telemedicine (60.94%), while 65.18% recognized that telemedicine could reduce visit time and 76.24% recognized its potential to reduce transportation costs. Prior exposure and use were less common, with 44.00% reporting that they had previously seen a telemedicine process and 44.71% reporting previous use of telemedicine services. These findings indicate that awareness of telemedicine and its potential benefits was more common than prior practical exposure or use.

**Table 3:** Respondents’ knowledge of telemedicine (*N* = 425).

Knowledge statement	Yes, *n* (%)	No, *n* (%)	Good knowledge
Have you heard the term telemedicine before?	259 (60.94)	166 (39.06)	Yes
Have you seen a telemedicine process previously?	187 (44.00)	238 (56.00)	No
Does telemedicine reduce visit time?	277 (65.18)	148 (34.82)	Yes
Does telemedicine reduce transportation costs?	324 (76.24)	101 (23.76)	Yes
Have you ever used telemedicine services before?	190 (44.71)	235 (55.29)	No

Table 4 summarizes respondents’ attitudes toward telemedicine. Mean item scores ranged from 3.21 to 3.42 on the five-point scale. The highest mean score was observed for the statement that telemedicine increases communication between patients and healthcare providers (3.42, SD 1.10), followed by improving access to healthcare services (3.41, SD 1.11). The lowest mean score was for the effectiveness of telemedicine for providing patient care (3.21, SD 1.20).

**Table 4:** Respondents’ perceived attitudes toward the adoption of telemedicine (*N* = 425).

Statement	Strongly disagree, *n* (%)	Disagree, *n* (%)	Neutral, *n* (%)	Agree, *n* (%)	Strongly agree, *n* (%)	Mean (SD)
Telemedicine is an effective tool for providing patient care	55 (12.94)	47 (11.06)	131 (30.82)	136 (32.00)	56 (13.18)	3.21 (1.20)
Telemedicine is cost-effective	34 (8.00)	52 (12.24)	130 (30.59)	148 (34.82)	61 (14.35)	3.35 (1.11)
Telemedicine can reduce unnecessary outpatient visits	37 (8.71)	56 (13.18)	95 (22.35)	182 (42.82)	55 (12.94)	3.38 (1.13)
Telemedicine can improve access to healthcare services	38 (8.94)	45 (10.59)	101 (23.76)	188 (44.24)	53 (12.47)	3.41 (1.11)
Telemedicine can be used for follow-up of patients with chronic conditions	31 (7.29)	55 (12.94)	106 (24.94)	177 (41.65)	56 (13.18)	3.40 (1.10)
Telemedicine increases communication between patients and healthcare providers	39 (9.18)	37 (8.71)	107 (25.18)	192 (45.18)	50 (11.76)	3.42 (1.10)
Telemedicine should be widely adopted in healthcare delivery	38 (8.94)	38 (8.94)	130 (30.59)	161 (37.88)	58 (13.65)	3.38 (1.11)

Table 5 reveals that respondents perceived multiple, fairly comparable barriers to telemedicine adoption, with mean scores clustering closely between 3.32 and 3.47 on a 5-point scale, indicating moderate-to-substantial agreement across all listed obstacles. Preference for face-to-face consultation emerged as the most salient barrier (mean = 3.47, SD = 1.17), closely followed by technological limitations (mean = 3.45, SD = 1.08) and patient resistance (mean = 3.41, SD = 1.10), suggesting that interpersonal and infrastructural factors weigh most heavily in respondents’ reservations toward telemedicine. Physician resistance and cultural aspects recorded identical mean scores (3.32), while diagnostic reliability concerns (mean = 3.37, SD = 1.09) also featured prominently, reflecting persistent apprehension regarding the clinical accuracy of remote consultations. The relatively uniform distribution of agreement across all six items, coupled with consistently high proportions of “agree” and “strongly agree” responses, indicates that barriers to telemedicine adoption in this population are multifactorial rather than dominated by a single overriding concern, encompassing provider-level resistance, patient attitudes, technological constraints, and sociocultural preferences simultaneously.

**Table 5:** Respondents’ perceived barriers toward the adoption of telemedicine (*N* = 425).

Statement	Strongly disagree, *n* (%)	Disagree, *n* (%)	Neutral, *n* (%)	Agree, *n* (%)	Strongly agree, *n* (%)	Mean (SD)
Physician resistance is a barrier to the practice of telemedicine	46 (10.82)	42 (9.88)	120 (28.24)	163 (38.35)	54 (12.71)	3.32 (1.15)
Patient resistance is a barrier to the practice of telemedicine	32 (7.53)	50 (11.76)	113 (26.59)	172 (40.47)	58 (13.65)	3.41 (1.10)
Diagnostic reliability is a barrier to the practice of telemedicine	33 (7.76)	46 (10.82)	134 (31.53)	153 (36.00)	59 (13.88)	3.37 (1.09)
Cultural aspects are a barrier to the practice of telemedicine	37 (8.71)	70 (16.47)	101 (23.76)	154 (36.24)	63 (14.82)	3.32 (1.17)
Technological limitations are a barrier to the practice of telemedicine	31 (7.29)	45 (10.59)	111 (26.12)	179 (42.12)	59 (13.88)	3.45 (1.08)
Preference for face-to-face consultation is a barrier to the practice of telemedicine	36 (8.47)	46 (10.82)	108 (25.41)	154 (36.24)	81 (19.06)	3.47 (1.17)

Table 6 presents the factors independently associated with telemedicine knowledge and attitude scores after multivariable adjustment. For knowledge, respondents aged 30–39 years had higher knowledge scores than those aged 18–29 years (*β* = 0.47; 95% CI: 0.10–0.84; *P* = 0.012). Higher knowledge scores were also associated with middle income (*β* = 0.72; 95% CI: 0.40–1.04; *P* < 0.001), high income (*β* = 0.62; 95% CI: 0.12–1.11; *P* = 0.015), chronic illness (*β* = 0.54; 95% CI: 0.22–0.85; *P* < 0.001), transport-cost barriers (*β* = 1.04; 95% CI: 0.72–1.36; *P* < 0.001), and social media account ownership (*β* = 0.64; 95% CI: 0.21–1.07; *P* = 0.003). In contrast, respondents from households with six or more family members had lower knowledge scores than those from households with five or fewer members (*β* = −0.38; 95% CI: −0.69 to −0.07; *P* = 0.018). Other variables, including sex, marital status, educational level, employment status, and smartphone ownership, were not significantly associated with knowledge scores after adjustment.

**Table 6:** Multivariable regression analysis of factors associated with telemedicine knowledge and attitude scores (*N* = 425).

Variable	Knowledge *β* (95% CI)	*P* value	Attitude β (95% CI)	*P* value
Age group				
18–29 years	Reference	—	Reference	—
30–39 years	0.47 (0.10, 0.84)	0.012	1.37 (−0.21, 2.96)	0.090
40–49 years	0.36 (−0.25, 0.96)	0.249	0.47 (−2.16, 3.09)	0.727
50–59 years	0.17 (−0.36, 0.70)	0.529	1.05 (−1.25, 3.35)	0.370
≥60 years	0.51 (−0.27, 1.30)	0.199	3.29 (−0.11, 6.68)	0.058
Sex				
Female	Reference	—	Reference	—
Male	0.02 (−0.26, 0.30)	0.894	0.69 (−0.52, 1.90)	0.262
Marital status				
Single	Reference	—	Reference	—
Married	0.08 (−0.30, 0.46)	0.679	3.56 (1.91, 5.21)	<0.001
Educational level				
No formal education	Reference	—	Reference	—
Primary education	−0.17 (−0.79, 0.44)	0.578	−0.28 (−2.95, 2.39)	0.838
Secondary education	−0.25 (−0.76, 0.26)	0.340	3.65 (1.44, 5.87)	0.001
University and above	−0.18 (−0.65, 0.28)	0.444	2.49 (0.49, 4.50)	0.015
Employment status				
Unemployed	Reference	—	Reference	—
Employed	−0.10 (−0.43, 0.23)	0.557	−1.15 (−2.57, 0.27)	0.111
Number of family members				
≤5	Reference	—	Reference	—
≥6	−0.38 (−0.69, −0.07)	0.018	0.81 (−0.55, 2.16)	0.241
Average monthly income				
Low	Reference	—	Reference	—
Middle	0.72 (0.40, 1.04)	<0.001	−0.30 (−1.69, 1.08)	0.669
High	0.62 (0.12, 1.11)	0.015	−1.04 (−3.18, 1.11)	0.343
Chronic illness				
No	Reference	—	Reference	—
Yes	0.54 (0.22, 0.85)	<0.001	−0.11 (−1.47, 1.25)	0.874
Transport cost barrier				
No	Reference	—	Reference	—
Yes	1.04 (0.72, 1.36)	<0.001	1.21 (−0.16, 2.58)	0.083
Smartphone ownership				
No	Reference	—	Reference	—
Yes	0.38 (−0.08, 0.84)	0.106	4.57 (2.57, 6.57)	<0.001
Social media account				
No	Reference	—	Reference	—
Yes	0.64 (0.21, 1.07)	0.003	0.14 (−1.70, 1.98)	0.880
Model fit statistics				
R2	0.306		0.174	
Adjusted R2	0.277		0.139	
F statistic (df)	10.53 (17, 407)	<0.001	5.03 (17, 407)	<0.001
Root MSE	1.39		5.99	
Observations	425		425	

For attitude scores, being married was associated with higher scores compared with being single (*β* = 3.56; 95% CI: 1.91–5.21; *P* < 0.001). Respondents with secondary education (*β* = 3.65; 95% CI: 1.44–5.87; *P* = 0.001) and university education or above (*β* = 2.49; 95% CI: 0.49–4.50; *P* = 0.015) also had higher attitude scores than respondents with no formal education. Smartphone ownership was strongly associated with more positive attitude scores (*β* = 4.57; 95% CI: 2.57–6.57; *P* < 0.001) and represented the largest coefficient in the attitude model. No statistically significant associations with attitude scores were observed for age group, sex, employment status, family size, income, chronic illness, transport-cost barriers, or social media account ownership.

Overall, the adjusted analysis identified different patterns of association for knowledge and attitudes. Knowledge was associated mainly with age, income, chronic illness, transport-cost barriers, family size, and social media access, whereas attitudes were associated mainly with marital status, educational attainment, and smartphone ownership. Both multivariable models were statistically significant. The knowledge model explained 30.6% of the variance in knowledge scores (R^2^ = 0.306; adjusted R^2^ = 0.277; F(17, 407) = 10.53; *P* < 0.001), whereas the attitude model explained 17.4% of the variance in attitude scores (R^2^ = 0.174; adjusted R^2^ = 0.139; F(17, 407) = 5.03; *P* < 0.001). Variance inflation factors ranged from 1.12 to 2.91 (mean 1.52), indicating no substantial multicollinearity.

## 4 Discussion

This community-based survey found moderate telemedicine knowledge among adults who participated in the study, with awareness of the term relatively common (60.94%) but previous exposure and reported use lower (44.00% and 44.71%, respectively). Attitudes toward telemedicine were generally favorable, with mean scores above the midpoint across all seven attitude items, particularly for improving patient-provider communication and access to healthcare services. Preference for face-to-face consultation, technological limitations, and perceived patient resistance were the most strongly endorsed barriers. In the adjusted analyses, age, income, chronic illness, transport-cost barriers, family size, and social media account ownership were associated with knowledge scores, whereas marital status, educational attainment, and smartphone ownership were associated with attitude scores. These findings provide community-level evidence on perceptions of telemedicine among adults reached through an online survey in Mogadishu. However, because recruitment used convenience and snowball sampling through online networks, the findings should not be interpreted as representative estimates for the wider Mogadishu population.

Approximately 61% of participants had heard of telemedicine, whereas only 44% reported having previously seen a telemedicine process and 44.71% reported previous use. This difference suggests that awareness of telemedicine was more common than direct exposure or reported use in the study sample. Similar variation between awareness and previous use has been described in studies from Egypt, Saudi Arabia, Ethiopia, and other settings (14, 16–18). Differences between settings may reflect variation in health-system experience, availability of telemedicine services, digital access, and exposure to remote-care platforms, although these explanations were not directly assessed in the present study (1, 19). Importantly, knowledge and attitudes in this study should not be equated with actual telemedicine adoption. Although previous use was assessed as part of the questionnaire, current or sustained adoption, frequency of use, type of service used, and continued intention to use telemedicine were not measured as primary outcomes. The findings therefore describe perceptions and reported previous exposure rather than population-level adoption.

Participants generally expressed favorable attitudes toward telemedicine, particularly regarding cost-effectiveness, access to healthcare, chronic disease follow-up, and communication with healthcare providers. However, the lowest mean score was observed for the perceived effectiveness of telemedicine for providing patient care (3.21, SD 1.20), with 30.82% selecting a neutral response. This may indicate that respondents were more confident about the potential logistical and communication benefits of telemedicine than about its effectiveness for clinical care. Comparable studies from Egypt, Saudi Arabia, and other settings have also reported favorable perceptions alongside concerns about the clinical applicability or reliability of remote consultations (14, 18, 20). In the present study, the generally favorable attitude scores indicate some openness toward telemedicine among respondents, but they do not establish willingness to adopt telemedicine at the population level or demonstrate that positive attitudes would translate into actual use. Further evidence from implementation studies would be needed to determine whether these reported attitudes correspond to uptake and sustained use.

The most strongly endorsed perceived barriers were preference for face-to-face consultation (mean 3.47), technological limitations (mean 3.45), and perceived patient resistance (mean 3.41), while diagnostic reliability concerns also received substantial agreement (mean 3.37). These findings suggest that respondents perceived multiple barriers operating at the patient, technological, and clinical levels rather than a single dominant obstacle. The preference for face-to-face consultation may reflect the continued importance of direct clinical interaction, whereas technological limitations are consistent with previously documented infrastructure and digital-access challenges in Somalia and other Sub-Saharan African settings (13, 19, 20). However, the item concerning patient resistance represents respondents’ perceptions of a potential barrier and should not be interpreted as direct evidence of resistance among patients. Similarly, the present study did not directly measure institutional trust, regulatory conditions, or provider competence; therefore, explanations based on these factors should be interpreted cautiously. The findings nevertheless indicate that addressing technological constraints, communicating clearly about the appropriate use and limitations of telemedicine, and engaging patients and providers may be important considerations in future implementation.

In the multivariable analysis, participants aged 30–39 years, those reporting middle or high income, participants with chronic illness, those identifying transportation cost as a healthcare-access barrier, and those with social media accounts had higher knowledge scores, whereas participants from households with six or more members had lower knowledge scores. These associations may indicate differences in exposure to health information, healthcare needs, or digital communication opportunities, but the cross-sectional design does not allow the direction or mechanism of these relationships to be established. For example, the association between transportation-cost barriers and knowledge may indicate that respondents experiencing greater access difficulties were more attentive to alternative forms of healthcare delivery, but it cannot establish that transportation barriers increased telemedicine knowledge. Similarly, the association between social media access and knowledge may reflect greater exposure to information through online networks, although the specific sources and content of such information were not assessed. The negative association observed for larger household size should also be interpreted cautiously because household resource sharing, digital access, health-information exposure, and other relevant factors were not directly measured.

For attitude scores, being married, having secondary or university-level education, and owning a smartphone were associated with more positive attitudes toward telemedicine. Smartphone ownership had the largest absolute coefficient within the attitude model, although this should not be interpreted as evidence of a causal effect. The association may reflect broader patterns of digital engagement, communication opportunities, or familiarity with mobile technologies. The relationship between education and attitude is also consistent with studies suggesting that digital familiarity and perceived usefulness may influence perceptions of telemedicine (21, 22). The Technology Acceptance Model and Diffusion of Innovation Theory provide potentially useful frameworks for interpreting these patterns; however, the present cross-sectional analysis was not designed to test these theories or establish causal pathways. The observed associations therefore indicate characteristics of respondents with more favorable attitudes rather than factors proven to determine telemedicine acceptance.

The findings have several practical implications that are directly linked to the variables assessed in this study. The combination of moderate knowledge and generally favorable attitudes suggests that public information and digital-health education may help improve understanding of telemedicine, particularly among groups with lower observed knowledge. Because technological limitations were among the most strongly endorsed barriers and smartphone ownership was associated with attitude scores, implementation efforts should also consider differences in access to appropriate devices and digital connectivity. The prominence of face-to-face preferences and concerns about diagnostic reliability indicates a need for clear communication about the types of health services that can appropriately be delivered remotely and the circumstances in which in-person assessment remains necessary. These measures should be evaluated through pilot implementation and subsequent assessment of actual uptake rather than assuming that favorable attitudes will automatically result in sustained telemedicine use.

### 4.1 Strengths and limitations

This study adds community-level evidence on telemedicine knowledge, attitudes, and perceived barriers in Mogadishu, where population-level evidence remains limited. The study included a relatively large sample and used a structured questionnaire that was adapted to the local context, translated into Somali, pilot-tested, and assessed for internal consistency. The inclusion of sociodemographic, healthcare-access, and digital-access variables and the use of multivariable regression allowed patterns of association with knowledge and attitude scores to be examined after adjustment for several measured characteristics.

Several limitations should be considered when interpreting the findings. First, the cross-sectional design establishes associations at a single period and therefore does not allow causal inference or determination of temporal relationships. Second, the use of online convenience and snowball recruitment through WhatsApp, Facebook, Telegram, and other social media platforms introduces a substantial risk of selection and coverage bias. Individuals who lacked internet access, did not use social media, had limited digital literacy, or were outside the recruitment networks had less opportunity to participate. The high levels of smartphone ownership, regular internet access, and social media use observed in the sample should therefore be interpreted as characteristics of the surveyed respondents rather than estimates for the wider Mogadishu population. In addition, because the number of people who received, viewed, or declined the survey invitation was not available, a conventional response or non-response rate could not be calculated, limiting assessment of possible non-response bias. Third, residual confounding cannot be excluded. Although the multivariable models adjusted for several measured sociodemographic and digital-access characteristics, potentially relevant factors such as digital and health literacy, trust in telemedicine, perceptions of privacy and confidentiality, previous experiences with healthcare, and other unmeasured socioeconomic or service-related characteristics were not fully captured. Associations therefore may partly reflect differences in these unmeasured factors. Fourth, the questionnaire was not previously validated. Although adaptation, translation, pilot testing, and internal consistency assessment were undertaken, these procedures do not establish full construct or criterion validity. The five-item knowledge measure may also not capture all dimensions of telemedicine knowledge. In addition, self-reported responses may be affected by recall and social desirability bias, particularly for previous exposure or use of telemedicine. Finally, the study was conducted only in Mogadishu and relied on an online non-probability sample. The findings should therefore be generalized cautiously to adults who can be reached through similar online networks and should not be extrapolated directly to rural populations, people with limited digital access, or other regions of Somalia with different healthcare and connectivity conditions. Despite these limitations, the study provides useful preliminary evidence that can inform future probability-based, mixed-methods, and longitudinal research examining telemedicine uptake and implementation in Somalia. In addition, the modest explained variance, particularly for the attitude model (adjusted R^2^ = 0.139), indicates that most of the variation in attitudes was not captured by the measured characteristics, further supporting the possibility that unmeasured factors play an important role.

## 5 Conclusion

Adults participating in this study demonstrated moderate knowledge and generally favorable attitudes toward telemedicine, while technological limitations, preference for face-to-face consultation, and perceived patient resistance were commonly reported barriers. Several sociodemographic, healthcare-access, and digital-access characteristics were associated with knowledge and attitude scores after multivariable adjustment. However, these findings should not be interpreted as causal relationships or as evidence of population-level telemedicine adoption because of the cross-sectional design and online non-probability sampling approach. The observed combination of favorable attitudes and identified barriers supports the need for targeted digital-health education, attention to technological access, and clear communication regarding the appropriate use and limitations of telemedicine. Future research should use more representative sampling and longitudinal or implementation designs to determine whether reported knowledge and attitudes translate into actual and sustained telemedicine use in Somalia.

## Data Availability

The original contributions presented in the study are included in the article/Supplementary Material, further inquiries can be directed to the corresponding author.

## References

[1] Dirie NI Ahmed MM Mohamed AA. Implementation of integrated telemedicine services in resource-limited Somalia: a case study of baano healthcare technology. Int J Telemed Appl. (2026) 2026:3667956. 10.1155/IJTA/3667956 41993706 PMC13081188

[2] WHO. Global strategy on digital health 2020-2025 2021. Available online at: https://www.who.int/publications/i/item/9789240020924 (Accessed July 18, 2026).

[3] WHO. The World Health Assembly endorses the extension of the Global Strategy on Digital Health to 2027 and approves the next phase for 2028–2033 (2025). Available online at: https://www.who.int/news/item/23-05-2025-world-health-assembly-endorses-extension-of-the-global-digital-health-strategy-to-2027 (Accessed July 18, 2026).

[4] Mehraeen E SeyedAlinaghi SA Heydari M Karimi A Mahdavi A Mashoufi M. Telemedicine technologies and applications in the era of COVID-19 pandemic: a systematic review. Health Informatics J. (2023):29. 10.1177/14604582231167431 PMC10116201 37076954

[5] Kruse C Heinemann K. Facilitators and barriers to the adoption of telemedicine during the first year of COVID-19. Systematic Review. J Med Internet Res. (2022) 24:e31752. 10.2196/31752 PMC8729874 34854815

[6] Agbeyangi AO Lukose JM. Telemedicine adoption and prospects in sub-sahara Africa: a systematic review with a focus on South Africa, Kenya, and Nigeria. Healthcare (Basel). (2025) 13:762. 10.3390/HEALTHCARE13070762 40218059 PMC11989057

[7] Wang M Huang K Li X Zhao X Downey L Hassounah S. Health workers’ adoption of digital health technology in low- and middle-income countries: a systematic review and meta-analysis. Bull World Health Organ. (2025) 103:126–135F. 10.2471/BLT.24.292157 39882495 PMC11774224

[8] Parkes P Pillay TD Bdaiwi Y Simpson R Almoshmosh N Murad L. Telemedicine interventions in six conflict-affected countries in the WHO eastern Mediterranean region: a systematic review. Confl Health. (2022) 16:64. 10.1186/S13031-022-00493-7 36517869 PMC9748883

[9] Bowsher G El Achi N Augustin K Meagher K Ekzayez A Roberts B. Ehealth for service delivery in conflict: a narrative review of the application of eHealth technologies in contemporary conflict settings. Health Policy Plan. (2021) 36:974–81. 10.1093/HEAPOL/CZAB042 33860797 PMC8355470

[10] Mahgoub EAA Nouri M. Navigating crisis: teleconsultation use and its determinants among internally displaced persons in wartime Sudan. BMC Health Serv Res. (2025) 25:1334. 10.1186/S12913-025-13430-0 41063140 PMC12509400

[11] Said AS Kicha DI. Implementing health system and the new federalism in Somalia: challenges and opportunities. Front Public Health. (2024) 12:1205327. 10.3389/fpubh.2024.1205327 38362207 PMC10867962

[12] Hassan MM Ali AN Ali I Mohamed ZO Abdullahi HM Ahmed MM. Regulation of health professions education and the growth of schools in Somalia. BMC Med Educ. (2024) 24:1178. 10.1186/S12909-024-06179-3 39427191 PMC11491018

[13] Garba B Jimale AO. Application of telemedicine to improve access, and quality of healthcare services in Somalia: a perspective review and policy recommendations. PAMJ. (2024) 49:100. 10.11604/PAMJ.2024.49.100.38299 40060278 PMC11889439

[14] Alboraie M Allam MA Youssef N Abdalgaber M El-Raey F Abdeen N. Knowledge, applicability, and barriers of telemedicine in Egypt: a national survey. Int J Telemed Appl. (2021) 2021:1–8. 10.1155/2021/5565652 PMC8192215 34211550

[15] Ahmed MM Othman ZK Sani J Mohamed AA Ali AN Aray AM. Knowledge, perceived benefits, perceived concerns, and predisposition to use telehealth services in Mogadishu, Somalia: a cross-sectional survey. Digit Health. (2025) 11:11. 10.1177/20552076251406298 PMC12696329 41393850

[16] Baradwan S Al-Hanawi M. Perceived knowledge, attitudes, and barriers toward the adoption of telemedicine services in the kingdom of Saudi arabia: cross-sectional study. JMIR Form Res. (2023) 7:e46446. 10.2196/46446 37315177 PMC10415944

[17] Shibabaw AA Chereka AA Walle AD Demsash AW Dube GN Dubale AT. Knowledge of telemedicine and its associated factors among health professional in Ethiopia: a systematic review and meta-analysis. PLoS One. (2024) 19:19. 10.1371/JOURNAL.PONE.0301044 PMC11025815 38635662

[18] Albaghdadi AT Daajani A Perceptions MM. Satisfaction, and barriers to telemedicine use: a community-based study from jeddah, Saudi arabia. Cureus. (2023):15. 10.7759/CUREUS.40738 PMC10361340 37485138

[19] Ahmed MM Dirie NI Mohamud AK Elmi AH Musa SS Alhammadi OAS. Advancing digital healthcare in Somalia: a review of modern technologies and their implications. BMC Digit Health. (2024) 2:49. 10.1186/S44247-024-00111-X

[20] Dodoo JE Al-Samarraie H Alzahrani AI. Telemedicine use in sub-saharan Africa: barriers and policy recommendations for COVID-19 and beyond. Int J Med Inform. (2021) 151:104467. 10.1016/J.IJMEDINF.2021.104467 33915421 PMC9761083

[21] Holtz B Mitchell K Hirko K Ford S. Using the technology acceptance model to characterize barriers and opportunities of telemedicine in rural populations: survey and interview study. JMIR Form Res. (2022) 6:e35130. 10.2196/35130 35436207 PMC9055487

[22] AlGhannam B Alsabah M Alainati S Alsaber A AlReshaid F AlHussainan O. Information technology acceptance and adoption in the telemedicine sector: a case of mobile health apps. Front Digit Health. (2026) 8:1718447. 10.3389/fdgth.2026.1718447 41778009 PMC12951316

